# Antiangiogenic Therapy as a New Strategy in the Treatment of Endometriosis? The First Case Report

**DOI:** 10.3389/fsurg.2021.791686

**Published:** 2021-12-06

**Authors:** Jean Bouquet de Joliniere, Arrigo Fruscalzo, Fathi Khomsi, Emanuela Stochino Loi, Floryn Cherbanyk, Jean Marc Ayoubi, Anis Feki

**Affiliations:** ^1^Department of Gynecology and Obstetrics, University Hospital of Fribourg, Fribourg, Switzerland; ^2^Department of General Surgery, University Hospital of Fribourg, Fribourg, Switzerland; ^3^Department of Obstetrics and Gynecology and Reproductive Medicine, Hôpital Foch, Suresnes, France

**Keywords:** endometriosis, VEGF, Bevacizumab, anti-angiogenesis, case report, Avastin

## Abstract

Angiogenesis plays a pivotal role in implantation and development of ectopic endometrial lesions. Thus, the potential usefulness of anti-angiogenic therapies has been speculated. Several reports describe their usefulness in animal models. Nonetheless this therapy has not been tested on humans yet. Here we report the outcome of a patient treated for a severe endometriosis with Bevacizumab (Avastin®), a monoclonal antibody directed against the vascular endothelial growth (VEGF). After a first-look laparoscopy with confirmatory biopsies was performed, three doses of Bevacizumab at 2-week intervals were administered. The therapy showed a well-tolerated profile and the prompt disappearance of the therapy-refractory chronic dysmenorrhea. A suppression of metabolic activity at the PET-scan compared to the basal one performed at diagnosis was also recorded. Furthermore, compared to the diagnostic biopsies prior the treatment, we documented a shift in the hormonal receptors profile toward a higher expression of progesterone and estrogen receptors in the endometriotic lesions.

## Introduction

Endometriosis is a benign disease characterized by ectopic endometrial implants outside the endometrium. It seems to affect about 5–10% of women of reproductive age, though its presence is often underestimated as clinical manifestations are heterogenic and diagnosis remains challenging (1). Importantly, if not adequately treated, endometriosis can develop over time into a potentially very disabling disease characterized by a wide spectrum of symptoms including severe chronic pelvic pain, subfertility or infertility as well as destruction of anatomical structures and organs, depending on site of implantation and extension (2, 3).

Current therapeutic options mostly rely on hormonal therapies, progestogen-only or combined estrogen-progestogen pills and GnRH analogs, administered to block ovarian function and avoid perimenstrual pain (4). Nonetheless, this conservative therapy can in some cases be unsuitable or even fail, necessitating the surgical removal of implants that implies the considerable intra- and post-operative issues linked to such a destructive surgery. This scenario occurs in most advanced forms of endometriosis linked to chronic and therapy-refractory pain and organ disruption (5).

Bevacizumab (Avastin®) is a monoclonal antibody directed against the vascular endothelial growth (VEGF) (6). As angiogenesis is one of the predominant factors involved in implantation and development of ectopic endometrial implants we speculated on its potential usefulness in the treatment of endometriosis (7). Animal experiments showed potential beneficial effects both in the therapy and in the prophylaxis of development of endometriosis-like lesions (8). Nonetheless, its potential usefulness on humans has not been tested to date.

Here we report the outcome of a patient affected by a severe form of endometriosis infiltrating the rectosigmoid and the pelvic wall and treated with Bevacizumab before surgical removal of the endometriosis nodule. The clinical, the metabolic activity at the PET-scan and the immunological profile at histochemistry before and after the adjuvant therapy have been evaluated.

## The Clinical Case

This case refers to a 46-year-old patient, admitted to emergency for acute pelvic pain with recto sigmoid mass and mechanical ileus. An abdominal CT scan performed in emergency showed colonic dilation with a stenosing tumor-like mass at the recto-sigmoid bordering the left ovary. The patient was hospitalized in the surgical department for clinical monitoring and further assessment. The next day, the patient developed an acute peritonitis requiring an emergency laparoscopy. In consideration of the suspected malignant origin of the mass stenosing the recto-sigma, the general surgeons performed a temporary transversostomy for bypassing the intestinal obstruction.

**Figure 1:**
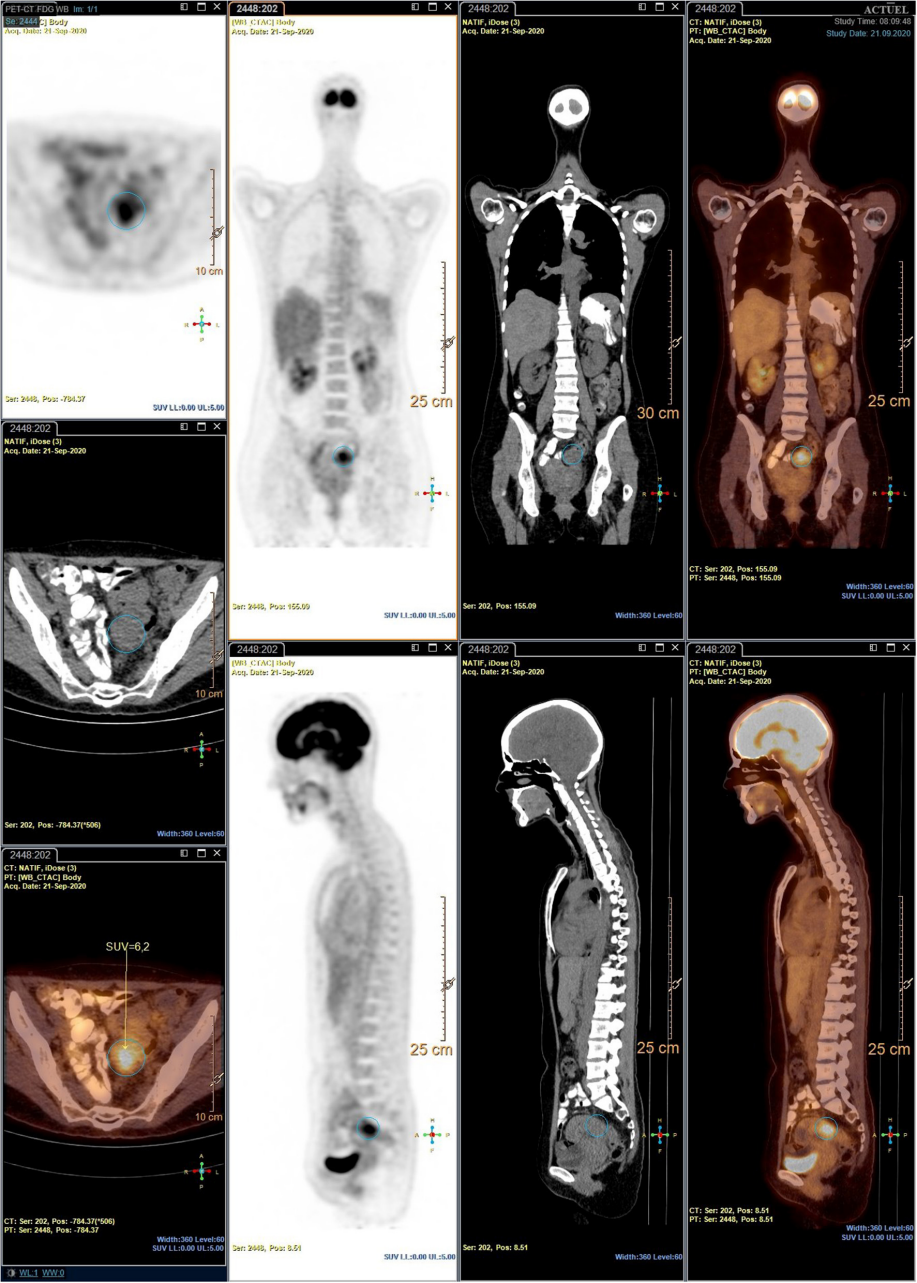
18F-FDG PET-CT, pre-treatment with Bevacizumab: intense recto-sigmoid hypercaptation (circled in blue) without further hypermetabolic lesions.

Subsequently the patient was discharged. A PET scan requested for differential diagnosis revealed a recto sigmoid hypercaptation, suggesting a primary intestinal tumor without evidence of lymph node metastases (Figure 1). A colonoscopy with biopsies was performed, showing a recto-sigmoidal stricture, without any evidence of cancerous lesions.

Thereafter, a diagnostic laparoscopy was performed, revealing a deep infiltrating endometriosis of the recto-sigmoid with involvement of the left pelvic wall and adnexa. The removal of the left adnexa for histopathology was undertaken, while the severity of the infiltration required the postponement of a curative surgical treatment. A series of further biopsies were taken for histological examination, which confirmed the diagnosis of endometriosis.

Faced with a positive anamnesis of invaliding dysmenorrhea despite the use of a combined oral contraceptive pill since the age of 25, the “off label” treatment with three doses of 5 mg/Kg intravenous Bevacizumab (Avastin®) at 2-week intervals to control pain relapse was proposed. No dyschezia, nor dyspareunia, chronic pelvic pain or menstrual abnormalities were reported in the anamnesis. An informed consent was signed. A good tolerability profile of Bevacizumab was observed, without any side-effects registered during and after its administration. Importantly, the complete disappearance of dysmenorrhea was observed at the end of therapy. A second PET-scan was then performed, highlighting the suppression of metabolic activity of the endometriotic nodule in the pelvis.

One month after the last dose of Bevacizumab, a second-look intervention with recto-sigmoid resection, removal of residual endometriosis and contralateral adnexectomy was performed. Macroscopically, endometrial lesions remained unchanged in dimension. However, they healed in a fibrous fashion that made dissection difficult. Histopathology confirmed a diffuse fibrosis. Interestingly, compared to the pre-treatment biopsies, a shift in the hormonal receptors profile toward a higher estrogen, progesterone and androgen receptors expression was highlighted (Table 1).

**Table 1:** Immunohistochemical characterization of endometriosis lesions.

	**Pre-treatment with Bevacizumab**		**Post-treatment with Bevacizumab**	
	**Endometrial stroma**	**Glandular epithelium**	**Endometrial stroma**	**Glandular epithelium**
ER	50%	60%	90%	100%
PR	85%	30%	90%	100%
AR	30%	5%	60–80%	90%
CD31	10–15%	0%	10–15%	0%
cMyc	30–40%	0%	<10%	60%
p53	70%	10–15%	70%	70%
Ki67	15%	0%	<5%	<5%
CD45	45%	0%	45%	0%
CD10	90%	0%	90%	0%

*ER, estrogen receptor; PR, progesteron receptor; AR, androgen receptor*.

Currently, at 6-month follow-up, the protective transversostomy has been removed and the patient is symptoms-free under a continuous combined hormonal substitutive therapy. Informed consent was obtained from the patient for the publication of this case report (including all data and images).

## Discussion

This is the first report of the use of Bevacizumab (Avastin®) in the treatment of a patient affected by endometriosis.

Bevacizumab is a monoclonal anti-angiogenic antibody directed against the VEGF. It thus has the potential to impact one of the most important features of endometriosis, the neo-angiogenesis. Keeping in mind this background, we proposed this therapy as off-label to a patient with a severe form of endometriosis, refractory to conventional therapies.

According to this report, a favorable effect on pain control and an excellent tolerability profile were obtained. Importantly, we observed an increased expression of hormonal receptor in endometriosis tissue. These results could be of high clinical relevance, knowing the central role played by progestins in inhibition of cell proliferation, inflammation and neovascularization (9). Reverting refractoriness to conventional hormonal therapy could help toward a conservative approach to the pathology.

Furthermore, we recorded a good profile of tolerability, without side effect. Nonetheless, due to its anti-angiogenic activity, Bevacizumab should be administered with caution, being linked to cardiovascular, renal and intestinal side-effects (10). A deficient wound healing linked to the treatment with anti-angiogenesis agents should be also kept in mind when planning a second-look surgery. Finally, according to our experience, an increased tissue fibrosis could impact the surgery. Less bleeding, but a greater difficulty of implants dissection due to fibrosis should be considered (11).

Current therapeutic options mostly rely on conservative hormonal therapies. Hereby, combined hormonal therapy and, especially, progestins seems to represent the cornerstone of medications, being also able to inhibit cell proliferation, inflammation, neurogenesis and neovascularization in endometriosis (12, 13). However, for yet unclear mechanisms, some forms seem to be characterized by progesterone resistance, being thus refractory to conventional medical therapy (14). In these cases, GnRH analogs could be proposed as second line therapy, but only for a limited period of time, due to their unfavorable side-effects profile (15).

Searching for new therapeutic options for refractory endometriosis to avoid destructive surgery should be a leading research topic for the future. In this prospective, the conservative treatment with Bevacizumab could be of interest where the conventional hormonal therapy failed, especially if progesterone and estrogen receptors are reduced or even absent. In this light, the results we present seem to be worthy of interest.

Animal experiments already showed beneficial effects both in the treatment and prophylaxis of endometriosis relapse (16–21). The effect of vascular endothelial growth factor inhibition on the volume of endometrial implants was described by all these authors. Furthermore, according to Ricci et al., Bevacizumab significantly inhibited cell proliferation in lesions, reduced vascular density and increased the apoptotic cell percentage (16). Also, Kebapcilar et al. showed higher reduction for the glandular epithelium and uterine vessels compared to controls treated with progesterone (18). Interestingly, Bevacizumab treatment did not affect ovarian reserve assessed by follicle quantification at histochemistry of ovarian serial sections (21).

However, even though these results in mice models were promising, potential usefulness of Bevacizumab on humans remains speculative at this stage. Indeed, the lesions created in rats were obtained from a transplant of endometrial tissue into the peritoneum and may not correspond to the pathophysiology of spontaneous disease in humans (22). Furthermore, vascular activity of endometriotic lesions could potentially affect the therapy efficacy. Indeed, endometriosis is a heterogeneous disease with different types of lesions, both the more active, red ones, as well as the older black and white ones (23). Recent research provided evidence for different patterns of vascularization, also for deep infiltrating bowel nodules. Interestingly, macroscopic features examined by using indocyanine green during surgery were consistently associated with characteristics of vascularization at histopathology (24, 25).

Thus, despite positive results recorded in this report, further studies should be performed to better evaluate clinical and biological results and define the optimal schema for treatment. Importantly, potential systemic negative effects of anti-angiogenic therapy should also be monitored, including ovarian reserve and fertility preservation. Once efficacy and safety of Bevacizumab treatment have been provided, clinical studies should evaluate its potential therapeutic impact in the future, also in the less advanced forms and in the prophylaxis of relapse of endometriosis.

## Conclusions

Fighting angiogenesis in cases of endometriosis refractory to conventional hormonal therapy seems a promising option to be evaluated in the near future. Further studies should be performed to better evaluate clinical and biological impact of this potential treatment option.

## Data Availability Statement

The original contributions presented in the study are included in the article. Further inquiries can be directed to the corresponding author.

## Ethics Statement

Informed consent was obtained from the patient for the publication of this case report (including all data and images).

## Author Contributions

JB and AFe: substantial contributions to conception, analysis, and interpretation of data. JB and AFr: drafting the article. JB, AFr, FK, ES, FC, JA, and AFe: substantial contributions to interpretation of data and revising the article for important intellectual content. All authors read and approved the final manuscript.

## Conflict of Interest

The authors declare that the research was conducted in the absence of any commercial or financial relationships that could be construed as a potential conflict of interest.

## Publisher's Note

All claims expressed in this article are solely those of the authors and do not necessarily represent those of their affiliated organizations, or those of the publisher, the editors and the reviewers. Any product that may be evaluated in this article, or claim that may be made by its manufacturer, is not guaranteed or endorsed by the publisher.
